# Focusing on Sodium Glucose Cotransporter-2 and the Sympathetic Nervous System: Potential Impact in Diabetic Retinopathy

**DOI:** 10.1155/2018/9254126

**Published:** 2018-07-05

**Authors:** Lakshini Y. Herat, Vance B. Matthews, P. Elizabeth Rakoczy, Revathy Carnagarin, Markus Schlaich

**Affiliations:** ^1^Dobney Hypertension Centre, School of Biomedical Science, University of Western Australia, Crawley, WA, Australia; ^2^Lions Eye Institute, Nedlands, WA, Australia; ^3^Dobney Hypertension Centre, School of Medicine, University of Western Australia, Crawley, WA, Australia; ^4^Department of Cardiology and Department of Nephrology, Royal Perth Hospital, Perth, WA, Australia

## Abstract

The prevalence of diabetes is at pandemic levels in today's society. Microvascular complications in organs including the eye are commonly observed in human diabetic subjects. Diabetic retinopathy (DR) is a prominent microvascular complication observed in many diabetics and is particularly debilitating as it may result in impaired or complete vision loss. In addition, DR is extremely costly for the patient and financially impacts the economy as a range of drug-related therapies and laser treatment may be essential. Prevention of microvascular complications is the major treatment goal of current therapeutic approaches; however, these therapies appear insufficient. Presently, sodium glucose cotransporter-2 (SGLT2) inhibitors may offer a novel therapy beyond simple glucose lowering. Excitingly, the EMPA-REG clinical trial, which focuses on the clinically used SGLT2 inhibitor empagliflozin, has been extremely fruitful and has highlighted beneficial cardiovascular and renal outcomes. The effects of SGLT2 inhibitors on DR are currently a topic of much research as outlined in the current review, but future studies are urgently needed to fully gain mechanistic insights. Here, we summarize current evidence and identify gaps that need to be addressed.

## 1. Diabetes and Its Complications

Diabetes mellitus is a chronic metabolic disorder marked by high blood glucose levels resulting from defects in insulin secretion, insulin action, or both. At present, diabetes is at pandemic levels. As per the International Diabetes Federation, in the year 2015, the global diabetic population was ~415 million. By the year 2040, this number is expected to increase to 642 million. All forms of diabetes can lead to the development of diabetes-specific macrovascular and/or microvascular complications. These macrovascular complications commonly affect the arteries of the heart (coronary artery disease), brain, and lower body whereas the microvascular complications can affect small blood vessels of the heart, kidney, foot, and the eye [1]. In this review, we will focus our attention on the microvascular complication of the eye which is known as diabetic retinopathy (DR).

## 2. Diabetic Retinopathy (DR)

Diabetic retinopathy is recognized as one of the most prevalent complications of both type 1 (T1D) and type 2 diabetes (T2D), and it is a leading cause of vision impairment [2]. Globally, the number of people with DR is estimated to grow from 126.6 million in 2011 to 191.0 million by 2030, and vision-threatening DR is projected to increase from 37.3 million in 2010 to 56.3 million by 2030 [3].

Retinal vascular abnormalities observed via ophthalmoscopy form the basis of the clinical diagnosis for DR [4]. However, a growing body of evidence supports that DR is both a vascular and a neurodegenerative disease of the retina [5]. Characteristic features of DR include hemorrhages, microaneurysms, and intraretinal microvascular abnormalities (IRMA) such as dilated preexisting capillaries, cotton wool spots, hard exudates (lipid deposits), retinal edema, and neovascularization [2, 6].

In general, DR is known to progress from a mild nonproliferative DR (NPDR) to a severe proliferative DR (PDR). The early stage of the disease often does not show recognizable retinal changes, leaving patients unaware of the condition. As retinopathy progresses, patients may invariably experience vision loss [6].

The early retinal change in DR is called nonproliferative or background DR. Nonproliferative DR can be subdivided into mild, moderate, severe, and very severe NPDR, according to the modified Airlie House classification of DR severity scale [4, 6]. During the NPDR stage, patients usually have no symptoms and have normal vision. Although the presence of microaneurysms is the hallmark of NPDR, histopathological observations of diabetic retinas show the presence of pericyte ghosts, acellular capillaries, and thickening of the capillary basement membrane [7] marking the mild or early NPDR stage. However, microaneurysms are the earliest opthalmoscopically detectable, clinical manifestation of DR. Typically, the pericyte to endothelial cell ratio is 1 : 1. Due to diabetes, pericytes necrotise significantly reducing the number of functional pericytes on capillary walls [7]. This selective loss of pericytes from the retinal capillaries is a characteristic lesion that occurs in the very early stages of DR. It is speculated that the loss of pericytes affects the integrity of the capillaries, leading to the formation of acellular capillaries, microaneurysms, or capillary occlusion [8]. Capillary occlusion is histologically manifested as an increased number of acellular capillaries. These capillaries are mere basement membrane tubes lacking endothelial cells and pericyte nuclei [9]. In addition, the mild NPDR stage can also manifest as hemorrhages and/or hard exudates [4, 6]. The presence of microaneurysms and retinal hemorrhages along with cotton wool spots or IRMA marks the moderate NPDR stage [4]. Moderate NPDR progresses to severe NPDR and shows microaneurysms with venous beading, hemorrhages, or both [6].

Cotton wool spots, also known as soft exudates, are round or oval spots with feathered edges. They appear white, grayish-white, or pale yellow-white under ophthalmoscopic observation. On the other hand, hard exudates are waxy, white, or yellow in colour with sharp edges. IRMA occur in intraretinal blood vessels between the arterioles and venules, closer to areas with capillary nonperfusion. The IRMA can be tortuous blood vessels formed due to intraretinal neovascularization or dilation of preexisting capillaries [10]. When very severe NPDR approaches, features of mild to moderate NPDR increase by severalfold in all four quadrants of the eye [6]. All microvascular changes of NPDR are limited to the inner retina and do not extend beyond the internal limiting membrane [11].

Proliferative DR occurs once NPDR progresses to its next level, with the growth of new blood vessels from preexisting vessels (neoangiogenesis) on the innermost layer of the retina. These blood vessels develop towards the vitreous body and its cavity. The neovascularization or formation of new blood vessels takes place in addition to the clinical features of NPDR. Extensive lack of oxygen leads to the development of neovascularization which is a random growth pattern of blood vessels. Winding of vessels and other various complicated formations indicates neovascular events. The newly formed preretinal vessels are thin, leaky, and fragile in structure and susceptible to hemorrhages, causing the leakage of blood into the vitreous body. As PDR progresses, fibrovascular scar tissue can form around new vessels. This tissue can be vascular or avascular. This fibrovascular variety can lead to retinal tearing and tractional retinal detachment followed by blindness [11]. The exact chronological sequence of development of the above lesions is still incompletely understood.

## 3. Molecular Aspects of the Pathogenesis of DR

One aspect of the pathogenesis of DR is linked to changes in retinal haemodynamics. The decreased blood flow through the retinal tissue leads to retinal hypoxia and has a significant impact on metabolic activities of the retina. The development of a hypoxic environment in the diabetic retina is evident prior to occurrence of other symptoms observed in diabetic patients [12] and diabetic animals [13] and is linked to capillary occlusion, a well-defined mechanism occurring in early DR [14]. As more capillaries become occluded with time, hypoxia in the surrounding retinal tissue enhances vascular endothelial growth factor (VEGF) expression [15]. This promotes breakdown of the blood-retinal barrier, increases vascular permeability, and stimulates endothelial cell growth and retinal neovascularization in the ischemic retina in PDR [16].

A number of other growth factors have been associated with the development of DR and these include platelet-derived growth factors (PDGFs), angiopoietin-1 and angiopoietin-2, basic fibroblast growth factor (bFGF), insulin-like growth factor-1 (IGF-1), stromal-derived factor-1, epidermal growth factor (EGF), transforming growth factor-beta 2 (TGF-*β*2), and erythropoietin. In addition to these, VEGF has been demonstrated as the main mediator of ocular neovascularization [16].

## 4. Perturbed Glucose Homeostasis and DR

Prolonged duration of diabetes and poorer glycemic and blood pressure control are strongly associated with DR. The storage of glucose in the retina is minimal compared to its high metabolic activity [17]. Thus, the retina is heavily dependent on the systemic circulation for the delivery of adequate glucose [18]. The endothelial cells of the capillaries of the inner blood-retinal barrier and the choroidal vessels across the retinal pigment epithelium (RPE) of the outer blood-retinal barrier are responsible for the transport of glucose across the retina. Excessive transport of glucose across the retina leading to high glucose concentrations within cells of the retina is a common factor leading to the pathogenesis of DR. Increased metabolic dysfunction due to high glucose levels and activation of signalling pathways as mentioned below lead to the development and progression of DR. Of the biochemical pathways involved, four major mechanisms have been found to explain the fate of cells and tissues exposed to hyperglycemia (Table 1). These include (1) activation of protein kinase C (PKC), (2) increased glucose flow via the polyol pathway, (3) increased advanced glycation end product (AGE) formation, and (4) increased hexosamine pathway flux [19]. All these pathways ultimately lead to increased oxidative stress, inflammation, and vascular dysfunction and upregulation of a wide range of growth factors and eventually contributes to the pathogenesis of DR.

At present, tight control of blood glucose levels is viewed as the primary means by which to disrupt the abovementioned pathways and slow the progression of retinopathy. However, the precise mechanism by which glucose enters the retina and how hyperglycemia causes retinopathy remains unclear. Therefore, in the past two decades, a major focus has been placed in understanding the role and mechanism of glucose transporters in DR.

## 5. Glucose Regulatory Mechanisms in DR

The entry of glucose into cells is regulated by facilitative glucose transporters (GLUTs) and sodium-dependent glucose cotransporters (SGLTs). In the sodium-dependent glucose transport process, SGLT1 and SGLT2 have been well characterised. In particular, SGLT1 is considered to be predominantly expressed in the brush border membrane of mature enterocytes in the small intestine and plays a major role in the absorption of glucose from the lumen of the intestine [20]. In contrast, SGLT2 is mainly found in the S1 and S2 segments of the renal convoluted proximal tubules and is required for the reabsorption of glucose [21].

The GLUT protein family is responsible for the glucose movement within the body, which occurs primary thought 14 isoforms [22]. Of these, GLUT1 and GLUT3 isoforms are expressed in a variety of tissue and cell types in the eye [23]. In particular, GLUT1 is expressed extensively throughout the eye and retina [24–26], suggesting GLUT1 as the main glucose transporter of the eye. In the retina, GLUT1 is highly expressed in pigment epithelial cells, Muller cells, and vascular endothelial cells forming the blood-retinal barrier [18, 25, 27, 28]. In contrast, relatively low levels of GLUT3 expression is seen in retinal inner and outer plexiform layers and pigment epithelium. In fact, it is predominantly expressed by the neuronal cells of the retina [26, 29–32]. Due to pathological characteristics of DR being associated with the loss of blood-retinal barrier function which leads to retinal microvascular abnormalities, GLUT1 and GLUT3 have been investigated in DR. Currently, the role of GLUT3 in DR is not well established. A recent study showed downregulation of GLUT3 in the retina of streptozotocin (STZ) diabetic rats with DR features and suggested this effect was a compensatory response to glucose during hyperglycemia. Furthermore, Knott et al. [32] reported upregulation of GLUT3 mRNA in cultured human retinal endothelial cells exposed to high glucose levels; however, these findings have been controversial due to the expression of GLUT3 being confined to the neurons of the retina [23]. The controversial nature of GLUT3 in DR and the limited number of studies that have investigated its role clearly show the need to further assess the role of GLUT3 in DR.

Given the entry of glucose into the endothelial cells of the inner blood-retinal barrier occurring primarily through GLUT1, its expression and glucose transport could have a major implication on the pathogenic pathways influencing the development and progression of DR. In retinal endothelial cells obtained from postmortem retinas from individuals with long-standing diabetes with minimal or no clinical features of retinopathy, it has been shown that upregulated GLUT1 is localised in the inner blood-retinal barrier. However, this upregulation of GLUT1 can serve to exacerbate the damage to the retinal microvasculature due to ongoing glucose influx [28]. Hypoxia which is a factor associated with the development of more severe retinal lesions in NPDR has been shown to increase GLUT1 expression in retinal endothelial cells [33]. Upregulation of GLUT1 mRNA has been reported in STZ-induced and galactose-fed diabetic rats with DR lesions [34, 35]. Furthermore, knockdown of GLUT1 by intraocular injections of siRNA after 2–4 weeks of STZ-induced diabetes in mice has shown restricted glucose transport by inhibiting GLUT1 expression, which decreased retinal glucose concentrations [36, 37]. In the same mouse model, blockade of GLUT1 reduced early biomarkers of DR such as superoxide radicals, chaperone protein b2 crystallin, and VEGF [36]. Taken together, these studies suggest that upregulation of retinal GLUT1 occurs early in DR and this plays a crucial role in disease progression.

**Figure 1:**
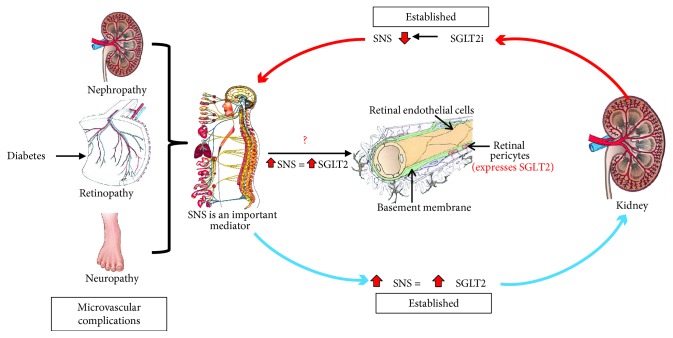
The potential molecular role of the SNS and SGLT2 in the pathogenesis of DR. Based on previous studies completed by our team and others, we propose a pivotal role for SGLT2 inhibition (SGLT2i) in the prevention of DR.

Interestingly and important in the current context, the expression of SGLT1 and SGLT2 (Figure 1) has been reported in the eye and the retina [24, 38, 39]. However, the full spectrum of SGLT expression and its role in the eye is poorly understood. Restricted expression patterns of both SGLT1 and SGLT2 have been reported in the lens [24]. It is suggested that SGLT1 is localised at the basolateral membrane of epithelial cells of the chick RPE [38]. The RPE is responsible for transportation of nutrients, ions, and water from the choroidal circulation to the photoreceptors of the retina. Hence, the RPE is essential for the integrity and survival of the neural retina and consequently visual function [40]. In recent years, alteration of both the structural and secretory functions of RPE has been found in DR [41]. The possible localisation of SGLT1 in the basolateral membrane may cause a transmembrane Na+ gradient to drive glucose into the RPE [42], and glucose would diffuse into the neurosensory retina via the apically located GLUT1. Alterations caused to this circuit by high concentrations of glucose may lead to inducible nitric oxide synthase (iNOS) causing nitric oxide-dependent oxidative damage in RPE cells [43]. Although the precise role of SGLT1 in the retina and retinal disease is not well-known, the above hypothesis warrants investigations to establish the role of SGLT1 in DR.

Wakisaka and Nagao first reported the expression of SGLT2 in cultured bovine retinal pericytes [39] highlighting the functional glucose sensor role of SGLT2 in the retinal microvasculature. Excessive Na+-dependent glucose entry causes intracellular swelling of pericytes [44] and leads to loss of contractile function, pericyte death, and retinal hyperperfusion. Overexpression of the extracellular matrix (such as fibronectin, collagen IV, and laminin) is associated with the development of basement membrane thickening followed by microvascular occlusion and retinal hypoperfusion [45]. This shift in retinal haemodynamics can bring about several downstream triggers of DR. SGLT2 inhibitors have shown to reduce pericyte swelling and overexpression of the extracellular matrix [45]. Arresting the haemodynamic shift early in DR is of significant importance for the development of effective therapeutics for DR.

A new class of antidiabetic drugs act by inhibition of SGLT2, which decreases the reabsorption of glucose from the renal proximal tubules. Hence, specific SGLT2 inhibition (SGLTi) leads to the initiation of glucose excretion via the urine (glucosuria) resulting in reduced blood glucose levels [46]. The SGLT2 inhibitors dapagliflozin, empagliflozin, and canagliflozin have shown to reduce glycosylated haemoglobin levels (HbA1c), fasting plasma glucose levels, blood pressure, and body weight [47–50]. Additionally, it has been shown that SGLT2 inhibitors have protective effects, targeting two of the main complications of diabetes which are cardiovascular disease and kidney-related complications [47, 48]. These outcomes have been assessed mainly in T2D; however, many studies are currently underway with T1D [51]. Takakura et al. showed for the first time the effect of ipragliflozin on the diabetic retina in spontaneously diabetic Torii fatty rats. In this study, diabetic rats treated with ipragliflozin demonstrated reduced prolongation of oscillatory potential in the electroretinogram, irregularities of the outer nuclear layers of the neural retina were absent, and ipragliflozin inhibited the progression of cataract formation in the lens [52]. The first patient-based study evaluating the impact of dapagliflozin on the retinal microvasculature showed lowered retinal capillary flow and prevented retinal arteriole changes. In this study, the placebo-treated diabetic group showed increased wall-to-lumen ratio indicative of retinal vascular hypertrophy, while the dapagliflozin-treated group showed no changes in wall-to-lumen ratio, indicating the positive effect of dapagliflozin in the prevention of vascular hypertrophy and remodelling [53]. In addition, evidence exists to highlight that dapagliflozin reduced the incidence of DR by 10% in human T2D cohorts [54]. One possible mechanism of SGLT2i in DR is that it results in blockade of the renin-angiotensin system, improving glycemic control and blood pressure lowering [55, 56]. A recent meta-analysis conducted by Tang et al. showed that the risk of DR with the treatment of SGLT2i was similar to that with placebo suggesting that SGLT2i did not increase the risk of DR [57]. Thus far, clinical evidence has shown a statistically insignificant beneficial effect. However, none of these studies systematically assessed the retinal vascular and neural pathology in detail during the development and progression of DR. This opens up an opportunity to test the effectiveness of SGLT2 inhibitors in mouse models with retinal and neural damage associated with DR (Figure 1), such as the Akita and Akimba mouse models [5]. The Akita and Akimba mouse models develop diabetes ~4–8 weeks of age, and these strains progressively develop characteristics of DR ultimately leading to retinal neovascularization, a hallmark feature of DR. The ultimate goal of effective DR treatments should ideally arrest the progression of the disease to PDR where neovascularization occurs. Therefore, the availability of well-characterised DR mouse models such as the Akita and Akimba will provide an ideal vehicle to assess the effectiveness of SGLT2 inhibitors in the pathogenesis and progression of DR. These studies are currently being conducted by our team.

## 6. Possible Interaction with the Sympathetic Nervous System

**Figure 2:**
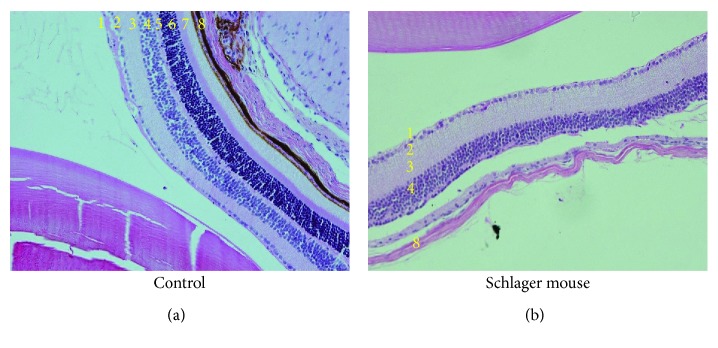
Sympathetic nervous system hyperactivation in the Schlager mouse is associated with neural damage of the outer layers of the retina. Haematoxylin and eosin staining of the eye of the Schlager mouse highlights that the outer plexiform, outer nuclear, and photoreceptor layers are all absent. 1: nerve fibre layer; 2: ganglion cell layer; 3: inner plexiform layer; 4: inner nuclear layer; 5: outer plexiform layer; 6: outer nuclear layer; 7: photoreceptor layer, and 8: choroid. Magnification: 200x.

Recent findings indicate that relevant interactions exist between SGLT2 expression and the sympathetic nervous system. Hyperactivity of the sympathetic nervous system (SNS) is a characteristic feature of obesity and T2D [58, 59]. We have shown for the first time that the major neurotransmitter of the SNS, noradrenaline, upregulates SGLT2 protein expression in human renal proximal tubule cells (Figure 1). In addition, high-fat diet-fed mice treated with dapagliflozin showed a reduction in tissue noradrenaline content and tyrosine hydroxylase expression in both heart and kidney tissue which points to a significant sympathoinhibitory action of SGLT2 inhibitors in our animal model [60]. To date, a limited number of studies have investigated the autonomic nervous system in relation to DR, and these studies suggest DR to be associated with early autonomic dysfunction in T1D and T2D patients [61]. The balance between sympathetic and parasympathetic effects determines the proper function of the autonomic nervous system. We have produced novel data highlighting that in a mouse model of neurogenic hypertension with a substantially activated SNS, neural damage of the outer layer of the retina is evident which is a characteristic feature of DR (Figure 2). We are now conducting mechanistic studies that aim to understand these preliminary findings. As we have already documented [60] that SGLT2 inhibition may downregulate the SNS in the heart and kidney, it is plausible that SGLT2 inhibition may also alleviate detrimental retinal changes that may be underpinned by hyperactivation of the SNS (Figure 1). Our recent results highlight an opportunity to investigate the role of SGLT2 and the SNS in DR.

## 7. Alternative Therapies for the Management of Diabetic Retinopathy

Controlling systemic factors such as blood glucose levels and blood pressure have shown to reduce the development and the progression of nonproliferative to proliferative DR.

Medical interventions such as the use of antiplatelet agents, PKC inhibitors, aldose reductase inhibitors, growth hormone/insulin-like growth factor, and intravitreal administration of corticosteroids and antiangiogenesis agents have proven to be effective at varying levels in limiting visual loss in DR. Among these, antivascular endothelial growth factor (VEGF) therapy administrated into the vitreous cavity represents one of the most beneficial treatment strategies implemented to treat PDR and diabetic macular edema (DME) [16].

Laser and surgical interventions such as panretinal laser photocoagulation and vitrectomy have proven to be effective in treating severe NPDR and PDR. In fact, until recently, panretinal laser photocoagulation was the first and only choice for treating PDR. Furthermore, vitrectomy has been used for the treatment of PDR with vitreous hemorrhages or fibrosis. Vitrectomy is currently used in treating ongoing DME. In the eyes with DME, focal laser treatment has been able to maintain visual acuity without future deterioration. Despite the availability of several treatments, DR remains a major clinical challenge with prevalence expected to rise further over the next decades. Alternative and preventative measures are therefore urgently needed. Based on the limited data available at this stage, therapeutic targeting of the SGLTs, particularly SGLT2, may provide a novel therapeutic opportunity to curb the global burden of DR. Similarly, targeting sympathetic hyperactivity per se and thereby its haemodynamic and inflammatory consequences may provide a useful novel target for DR. Relevant studies are currently being conducted and may well provide further support for such an approach in due course.

## Figures and Tables

**Table 1:** Proposed mechanisms involved in the pathogenesis of DR. Prolonged exposure to hyperglycemia leads to the activation of a number of interconnecting biochemical pathways that contribute to the pathogenesis of DR.

Pathway	Mechanism	Reference
Activation of protein kinase C (PKC)	(i) Hyperglycemia increases the synthesis of diacylglycerol (DAG). (ii) Several PKC isoforms (such as PKC-*β*, *α*, and *δ*) are activated. (iii) Phosphorylation of substrate proteins mediated via PKC promotes changes in retinal blood flow, increased vascular permeability, endothelial cell dysfunction, and altered growth factor signaling. (iv) Retinal ischemia and neovascularization result.	[62]

Polyol pathway	(i) Metabolizes excess glucose to sorbitol and then fructose during hyperglycemia. (ii) Intracellular accumulation of sorbitol leads to osmotic stress in retinal cells including ganglion cells, Muller glia, vascular endothelial cells, and pericytes. (iii) Pathophysiological consequences include thickening of capillary basement membrane, pericyte loss, formation of acellular capillaries, microaneurysms, hemorrhages, glial cell activation, and apoptosis.	[63]

Advanced glycation end product (AGE) formation	(i) Hyperglycemia increases the formation of AGEs. (ii) Production of AGEs damages target cells via three mechanisms: (1) Changed extracellular matrix brings about abnormal interactions with matrix receptor proteins and surrounding matrix components. (2) AGE-modified intracellular proteins have altered functions. (3) AGE-modified plasma proteins bind to the receptor for advanced glycation end products (RAGE) on endothelial cells, leading to the production of reactive oxygen species.	[64]

Increased hexosamine pathway flux	(i) During hyperglycemia, ~3% of glucose is processed through the hexosamine pathway. (ii) Fructose-6-phosphate is converted to glucosamine-6-phosphate. (iii) Subsequently, glucosamine-6-phosphate is metabolized to UDP-GluNAc (uridine diphosphate N-acetyl glucosamine). (iv) UDP-GluNAc attaches to serine and threonine residues of transcription factors.	[65]
